# The potential role of veterinary technicians in promoting antimicrobial stewardship

**DOI:** 10.1186/s12917-023-03637-w

**Published:** 2023-09-02

**Authors:** Laurel E. Redding, Katherine Reilly, Bridget Radtke, Stacy Bartholomew, Stephen D. Cole

**Affiliations:** 1 Department of Clinical Studies-New Bolton Center, School of Veterinary Medicine, University of Pennsylvania, Kennett Square, Philadelphia, PA USA; 2 Department of Pathobiology, School of Veterinary Medicine, Philadelphia, PA USA; 3 Department of Veterinary Technology, Manor College, Jenkintown, PA USA

**Keywords:** Antimicrobial stewardship, Antimicrobial Resistance, Veterinary technicians, Veterinary nurses, Qualitative study, Engagement

## Abstract

**Background:**

A core principle of antimicrobial stewardship (AMS) in veterinary settings is the need for engagement of all stakeholders; however, no studies have addressed the role of veterinary technicians in AMS specifically. The objective of this study was to qualitatively assess knowledge, opinions, and practices related to AMS among technicians. Semi-structured interviews were conducted with 33 veterinary technicians with varied backgrounds, experience and roles. Interviews centered on participants work experience and interactions with their employer, perceptions of antimicrobial resistance and overuse in veterinary medicine, observed application of AMS principles, opinions on potential opportunities for technicians to contribute to AMS and concomitant potential barriers to these opportunities. Transcripts of interviews were coded thematically by two authors, then organized into a hierarchical framework, and the characterization of codes was compared across different categories of respondents.

**Results:**

Most veterinary technicians were knowledgeable about antimicrobial drugs but could not provide a complete definition of antimicrobial resistance or AMS. Most veterinary technicians could identify examples of antimicrobial misuse. Participants identified areas of client education and discussion with veterinarians as potential areas to contribute to AMS. Barriers identified included hierarchical structures of veterinary practices and time-constraints. Most participants expressed a personal interest in participating in AMS.

**Conclusions:**

There is a possible appetite among some veterinary technicians to participate in AMS and they already play applicable roles in practices. Barriers such as educational needs, hierarchical structures of veterinary practices and time constraints will need to be addressed if technicians are included in AMS efforts.

**Supplementary Information:**

The online version contains supplementary material available at 10.1186/s12917-023-03637-w.

## Background

Antimicrobial resistance is one of the most significant challenges that both veterinary and human medicine face today [1]. Antimicrobial stewardship (AMS) is defined by the American Veterinary Medical Association (AVMA) as “the actions veterinarians take individually and as a profession to preserve the effectiveness and availability of antimicrobial drugs through conscientious oversight and responsible medical decision-making while safeguarding animal, public, and environmental health”[2]. Antimicrobial stewardship programs are increasingly being deployed in veterinary settings, both at the individual and institutional levels [3–8]. Historically, efforts to understand, measure and alter antimicrobial use has primarily focused on production animal sectors; however, in recent years there has been increased focus on companion animal medicine [2, 4–6]. In the US, use of antimicrobials is not as heavily regulated as in food-producing animals [2]. Concerns over the emergence in antimicrobial resistant bacteria among companion animals are not only important to the medical outcomes for individual pet infections, but also because it has been demonstrated that antimicrobial-resistant bacteria can be shared between pets and owners [7–10].

One of the AVMA’s core principles of AMS is to engage all practice members in stewardship efforts, including veterinary paraprofessionals such as veterinary nurses, technicians and assistants [2]. In human medicine, recent work has highlighted the critical roles that nurses can play in AMS [11]. Specific tasks traditionally performed by nurses that impact antimicrobial prescribing and stewardship include collection of specimens for microbiology testing, administration of antimicrobials, monitoring patients for adverse effects, providing education for home-care, and advising on intravenous-to-oral (IV-to-PO) conversion [12, 13]. Veterinary nurses, technicians, and assistants perform similar roles as human nurses and could therefore potentially plan an important role in veterinary AMS. Studies have demonstrated enthusiasm among human nurses to participate in AMS, but also a need to address barriers and provide relevant training [13, 14].

Very little has been articulated about the potential role of veterinary nurses, technicians, or assistants in AMS, and no studies have assessed their interest in or ability to promote AMS. The goal of this study was therefore to qualitatively assess knowledge, opinions, and practices related to antimicrobial use and stewardship in a diverse group of veterinary paraprofessionals, with the ultimate goal of informing future policies and practices relating to AMS that can be undertaken or supported by veterinary technicians across diverse veterinary practices.

## Results

### Participants

A total of 25 certified veterinary technicians (CVT- defined as credentialed individuals with state licensures) and 8 veterinary assistants (individuals without state licensure) were interviewed. At the beginning of the study, the study team approached 10 key contacts within their professional network which yielded at least 1 participant per contact. Given the open style of recruitment (e.g. use of both listservs, social media posts, recruitment via information sharing) it is not possible to characterize the population of individuals who chose not to participate in the study. Job titles are often used interchangeably throughout the field and the term “veterinary technician” is used throughout this manuscript as a catch-all term. Most of the participants were from the northeastern United States (Table 1). All participants except one were female, and most (n = 26, 78.8%) worked exclusively with small animals (Table 1), which is reflective of the population of veterinary technicians in the United States [15]. A majority of participants (n = 20, 60.6%) worked at an academic institution or referral practice, while approximately a third (n = 11, 30.3%) worked at a general practice. Given that most of our participants identified themselves as a “veterinary technician”, we will use the term “veterinary technician” throughout the rest of the manuscript to encompass all participants. The authors acknowledge and respect that different terms are used throughout the field, and more distinct definitions are needed.

**Table 1:** Participant demographics

Age (mean (SD) years)	37.6 (9.5)
Sex – n (%)	
Female	32 (97.0)
Male	1 (3.0)
Highest degree obtained - n (%)	
General Educational Development	3 (9.1)
certificate	
Associates degree	17 (51.5)
Bachelors degree	12 (36.4)
Masters degree	1 (3.0)
Mean (SD) number of years working as a veterinary nurse/technician/assistant	13.6 (8.5)
Type of practice at which participant is employed – n (%)*	
General private practice	11 (30.3)
Academic institution	9 (27.3)
Referral practice	11 (33.3)
Shelter	1 (3.0)
Lab animal	1 (3.0)
Zoo	1 (3.0)
Types of species worked with – n (%)	
Small animal exclusively	26 (78.8)
Large animal exclusively	2 (6.1)
Mix of small animal and large animal	2 (6.1)
Mix of small animal and exotics	3 (9.1)
Zoo/exotics exclusively	1 (3.0)
Location (state)	
Pennsylvania	17 (51.5)
New Jersey	7 (21.2)
Illinois	6 (18.2)
New York	1 (3.0)
Rhode Island	1 (3.0)
Washington DC	1 (3.0)

*Some technicians worked at more than one place, which is why the total number exceeds 33

Demographic data for the 33 veterinary technician antimicrobial stewardship interview participants

All participants reported being involved to some extent in routine tasks that may have an impact on antimicrobial use at their practice (i.e., every participant was routinely engaged in at least one of the following tasks, and many in most or all of them). Tasks included: collecting patient medical histories which provides critical information such as vaccination status for infectious diseases and previous antimicrobial use, reporting on patient progress to the veterinarian including clinical response (or lack thereof) to antimicrobial administration, calculating doses of and administering antimicrobial drugs in an accurate and timely manner, discussing therapeutic plans with owners including proper antimicrobial administration strategies, participating in infection control measures for the mitigation of nosocomial infections which may require subsequent antimicrobial therapy, and teaching other veterinary technicians about the aforementioned tasks and bacterial infections. These tasks were included because they either fell within previously described AMS duties for human nurses or are part of antimicrobial stewardship checklist created by the AVMA or both [2, 12, 13].

### Knowledge of antibiotics

All participants reported that antibiotics were prescribed regularly in the practices they worked at, and on average, 5.1 of every 10 patients seen in their practices were prescribed antibiotics. When asked to define an antibiotic, most participants were able to give a complete or partially complete definition (i.e., some element of the definition “a medication that kills or stops the growth of bacteria [16]. When asked how antibiotics are used in the veterinary profession in general, approximately half of the participants believed they are generally used responsibly. Others believed antibiotics were or used to be used inappropriately and cited examples of inappropriate use, including prescribing them when other treatments or no treatments would suffice, prescribing unnecessarily in response to clients demands or finances, prescribing without appropriate diagnostics (e.g., culture, cytology), using the wrong drug, and the client using the drug incorrectly (Table 2). When asked to define antibiotic resistance (i.e., “some variation of a genetic mechanism inherent to or acquired by bacteria to evade the effects of antibiotic drugs), 11/33 (33.3.%) were able to provide a complete definition, while the remaining provided partial definitions. Note that during interviews with respondents, the term “antibiotic” was consistently used.

**Table 2:** Veterinary technicians’ perceptions on antibiotic use and antimicrobial stewardship in the veterinary profession

Theme	Illustrative quote
**Antibiotic use in the veterinary profession**	
Antibiotic use is generally responsible (n = 15)	“The practices I’ve worked at have been really responsible with their medical policies and protocols […] and they make sure they do their due diligence.”
Antibiotic use is generally irresponsible (n = 13)	“I think we don’t have a great handle on how often they’re being used, and I think they’re being used a lot more frequently than they really truly need to be. I think that there’s just a lack of education about why we maybe shouldn’t be using all of these antibiotics all the time.”
Antibiotic use is becoming more responsible (n = 5)	“So I feel like when I was first started out, antibiotics were given much more than they are now. […] [I] think they are beginning to be not as excessive. I feel like before they were excessive and now they’re starting to I don’t know if it’s teaching differently or not, but I feel like they’re starting to give them appropriately”.
**Examples of inappropriate prescribing**	
Prescribing antibiotics when other treatments or no treatment would suffice	“We see a lot of patients with subclinical UTIs where they ran a UA because it’s part of their […] general wellness and then somebody starts them on an antibiotic despite them not having any clinical signs of an infection. And we’re finding that there’s really no reason to treat those guys unless they’re showing signs, but we see that every single day.”
Prescribing to accommodate clients’ demands or finances	“Sometimes I do feel that they’re being prescribed only to make the client happy. To appease the client. Especially with covid and the clients have been more angry at a base level. You know so maybe they don’t have the money to do a urinalysis. But you know what. They just want an antibiotic and I feel like sometimes I have seen veterinarians prescribe an antibiotic and say “Hey if it’s not better, you know, in three to five days or it’s not improving, give us a call back”, which I hate. Because we don’t hear back from those people, and then they get what they want and it’s not the right thing.”
Prescribing without appropriate diagnostics	“You’re using an antibiotic to treat something that you don’t have a diagnosis for. Kind of an assumed issue that, you know, sometimes you see are treated with an antibiotic plus or minus steroid just to see if it gets better. Sometimes that feels a little unnecessary if another step could be taken to get a diagnosis first.
**Examples of how antimicrobial stewardship is practiced within clinical practices**	
Using antimicrobials only when indicated	“We [the veterinary clinic team] try to balance, and you know we talked about this a lot actually in our rounds with regards to antibiotics and even with other type of things, but specifically we really tried to you know prescribe what’s needed. But we don’t want to you know overkill and prescribe more often than needed.”
Using appropriate diagnostics	“Performing a culture to ensure that the goal that they’re trying to achieve is completed appropriately so really determining what bacteria is there before we go ahead and give the antibiotic that will work against it.”
Discussion how to improve antimicrobial use in general within the practice	“I’ve definitely overheard our vets explain the concept of stewardship without using those specific words to our students and I feel like when there are the referrals that we feel like haven’t been treated as appropriately as they could be the other nurses and I do get together and are like ‘they could have done this better’ and kind of discuss that how it could have been improved.”

### Knowledge of antimicrobial stewardship

Most of the participants had not heard of the term “antimicrobial stewardship”. However, when provided with the definition articulated by the AVMA (i.e., “Antimicrobial stewardship refers to the actions veterinarians take individually and as a profession to preserve the effectiveness and availability of antimicrobial drugs through conscientious oversight and responsible medical decision-making while safeguarding animal, public, and environmental health. In a nutshell, it’s veterinarians and veterinary institutions making conscious efforts to prescribe antimicrobials appropriately to limit antimicrobial resistance” [2, 16]), most stated that AMS was practiced in some form at their clinic, though some said it was only “sort of” practiced, and a few said it was “not really” practiced. Examples of ways AMS was practiced included using antibiotics only when indicated, performing appropriate diagnostics (e.g., culture, cytology), discussions amongst staff on how antibiotic use can be improved within the practice, discussion on judicious antibiotic use with clients, stopping or changing antibiotics when necessary, ensuring rechecks and in-house monitoring of patients, and prioritizing topical medications (Table 2). Amongst technicians specifically, AMS was practiced by discussing treatment plans with veterinarians, educating clients about treatment plans, and teaching students and other technicians about judicious use of antibiotics.

### Technician involvement in antimicrobial stewardship

When asked how veterinary technicians could participate in AMS, respondents identified a variety of ways (Table 3). A primary role was to participate in discussions with veterinarians about treatment plans: technicians could provide input on the patient’s condition to aid in making treatment decisions and act as patient advocates, especially in the case of patients with highly dynamic clinical conditions, where they are often the ones monitoring the patient most closely. In referral institutions, some veterinary technicians thought they could have a role to play in facilitating dialogue about a patient amongst different team members/specialties to optimize clinical decision making. Another important way technicians described being able to contribute to AMS was in providing client education: explaining why drugs are needed (or not needed), how to administer them appropriately, and conveying the importance of follow-up visits. Respondents reported that many of their typical duties within the clinic contributed to AMS efforts, including taking thorough patient medical histories, participating in infection control, appropriately filling medication orders, and monitoring patient progress. Finally, respondents saw a role in educating other technicians and students in concepts of judicious antibiotic use.

**Table 3:** Roles for, barriers to and facilitators of veterinary technician and assistant involvement in antimicrobial stewardship

Theme	Illustrative quote
**Potential stewardship roles for veterinary technicians**	
Having discussions with veterinarians (n = 25)	“Having a conversation with the veterinarian and asking them why they’re choosing this versus something else […] because sometimes they will talk to like an internal medicine specialist or a criticalist, and they will come to that conclusion themselves, but we just need to know that that has happened and they’re not just doing this because that’s what usually happens.” “Advocating for our patient […] if we really don’t think they need or really do think they need something. Really just trying to communicate with the doctors to be, ‘hey I think they really need this antibiotic’ or ‘is this actually doing anything, nothing has changed in their status, why are we still continuing’?”
Client education (n = 25)	“I think that [educating clients] is something we could do more, I don’t hear a lot of like explanations about why we need these recheck, certain things were just like ‘oh and come back’. And then […] since that importance isn’t passed along as well as it could be, then we get a lot of […] no shows, and like ‘oops we missed that appointment we’ll do it in another two weeks’ and i’m like ‘well, now it’s been four weeks’ and…I feel like that could be impressed upon owners better.”
**Barriers to involvement in antimicrobial stewardship**	
Hierarchy in the workplace (n = 16): veterinarian-technician	“The concern there is that […] a lot of veterinarians, at least in my experience in surgery, aren’t interested in hearing what their nurses have to say. […] So there’s some some standoffishness there I guess.” “It would be very difficult, because we are not, you know, diagnosticians and we are not primarily responsible for diagnosis and treatment of patients. We’re here to carry out orders and recommendations by the veterinarians that are in charge of the patient.” “At this point in my career and [with] the doctors that i’m working with at this time, I would not probably ever feel comfortable [with me speaking up about antibiotic overuse]. In my past I’ve had doctors that I definitely would have felt comfortable saying ‘hey, you ever worry, you know, in a way, like do you ever worry that like maybe this is, it could lead to overuse or you know something’ and there’s people that would have received it well, but I don’t think it would be received well now.”
Hierarchy in the workplace (n = 16): client-technician	“You know, how the client perceives you. And I […] was in derm six years, I knew a lot of information, but they still wanted to talk to the doctor and the doctor could literally say exactly what I said, word for word. But they wanted to hear it from that person, so I guess the perception of a nurse versus doctor is a hard barrier to break.”
Time constraints (n = 13)	“Everything is so fast paced, you want to try to get people in and out, jumping to antibiotics is very easy. And so I think, having them kind of stop, slow down, think if that’s what they really want to use or do, or talk to the doctor about, I think that would be hard for people to change.”
Lack of interest (n = 8)	“My truthful answer is I don’t think [technicians] really care, to be honest with you, you know as much as it probably hurts you guys. I think there’s certain things in our field that people will think it’s just like “roll with it”, like it’s written on the thing, I get the drug and I administer it. It’s not something that they’re like ‘hey, you know, wait a minute, maybe we should like change this or stop it’, or you know, like I don’t I think it’s just become such a routine like just a treadmill type thing they just keep going and like nobody really stops to think about it.”
Lack of knowledge about antimicrobial stewardship (n = 7)	“I don’t think [technicians] enough know about [antimicrobial overuse], to know that they should be concerned. A lot of places have the same protocol it’s been the same protocol for a long time, as far as they know it works, so why change it. […] So I think if more knew about the importance of it, they would definitely be more interested… But if they don’t know that they should be advocating they don’t know that they should know, then that’s hard.”
Lack of confidence in their abilities - by themselves or the veterinarian (n = 10)	“I think that unfortunately GP just doesn’t elevate the certified techs…I do think that the specialties take more into account that they have a CVT and they try to elevate them and have us work to the top of our license, where kind of at GP you just get thrown in and you’re doing the same job that an assistant is doing…we go to school and we learn a lot in those two years, but yeah, just elevating them and actually using them for, you know, what they can help a doctor with” “I know a lot of new nurses, sometimes are still trying to learn the ropes so they’re focusing on like the hands-on stuff first”
**Ways to promote technicians’ engagement in antimicrobial stewardship**	
Regular discussions within the practice (n = 18)	“I think an open discussion is really critical to have. I hate the us-and-them, like with vets and vet nurses. It’s a team, we’re nothing without each other, so I definitely think it’s it should be an open conversation if [an antimicrobial] is being prescribed. As long as the nurse is approaching the question appropriately, I think the veterinarian should be open to that discussion. And then be able to explain that and have a conversation about it, because the nurse is interested.”
Presentation of educational material on stewardship (n = 9)	“I think, at least for me from a training standpoint, […] I think finding a way to make it interactive so finding you know either utilizing you know zoom features and breakout rooms and having dialogue and discussion. Because it’s one thing to present material, which I think is important to get everybody at a level setting place, but then have that discussion and dialogue and and even at that point, finding out what additional things vet nurses are looking for or need support with.”
Empowering technicians with responsibilities (n = 6)	“I think it depends on how a technician is used, I do know that there are practices where technicians are used more as assistants so they’re like restraining and that sort of thing, and then I do know there are places where technicians do have a lot of power. […] I think those ones will probably be interested [in promoting stewardship]”.
Frame stewardship as patient care (n = 4)	“I think if [stewardship] was something that was better presented in a way that like ‘this really does make a difference and we can make things go poorly when we don’t pay attention to this’. I think presented in that light, I think a lot more nurses would be more willing to learn about it.”

*Identified themes within veterinary technician antimicrobial stewardship interview transcripts with illustrative quotes from participants*

### Barriers to technician involvement in antimicrobial stewardship

While mostly expressing interest in AMS, veterinary technicians acknowledged substantial barriers to being able to do so (Table 3). The main barriers were hierarchy and time constraints. Because of the veterinarian-technician hierarchy, many technicians did not feel they were able to advise veterinarians or question their prescribing decisions, even if they disagreed with them, either because they perceived that veterinarians may not be interested in their opinion or that the veterinarian would not react positively to a suggestion from a veterinary technician regarding antibiotic therapy. There was also a perceived hierarchy with regards to the client, where technicians felt like they could not educate clients because clients only listened to the veterinarian. Time constraints were also major barriers: technicians felt like they already did not have enough time to accomplish everything that needed to be accomplished within an appointment and that adding AMS efforts would be prohibitive. Some respondents cited a lack of interest in AMS, especially among non-CVT technicians, as a barrier to stewardship, while others cited a lack of knowledge about AMS or a lack of confidence in their abilities (either by themselves or the veterinarian) as reasons for not being able to be involved in stewardship.

### Appetite for involvement in antimicrobial stewardship

When asked if there was an appetite for learning more about and becoming involved with AMS among veterinary technicians in general, most respondents thought there was, while others thought there were too many barriers (Table 3). A majority of participants indicated they personally would want to become involved in AMS, and certain respondents noted that there were benefits to being involved in AMS, including improving client compliance and satisfaction, empowering technicians, and lessening the workload of veterinarians. A common observation was that there was more appetite for and ability to participate in AMS in specialty or academic practices than in general practice. Participants thought engagement in AMS could be facilitated in a variety of ways, including holding regular discussions on the topic within the practice, the presentation of engaging educational material on AMS, empowering technicians with greater responsibilities, and framing AMS as patient care (Table 3). Respondents independently suggested that education on the topic could be achieved by on-the-job training, continuing education, workplace presentations (e.g., “lunch-and-learns”), and integration into the CVT curriculum.

### Critical points for technicians to know about antimicrobial use and stewardship

Following the end of the interview, once the issue of AMS had been defined and discussed, respondents were asked to articulate the most important “take-home” messages about antibiotic use and antimicrobial stewardship. Respondents cited the following six points:

- Knowing what antimicrobial resistance is and how it develops.
- Understanding how antibiotics work.
- Antibiotics should only be used when indicated.
- The importance of performing appropriate diagnostics (e.g., culture and sensitivity testing).
- The importance of being informed and communicating about AMS.

## Discussion

In this study, we employed a qualitative approach to characterize the knowledge, opinions and practices of veterinary technicians surrounding AMS. While some of the veterinary technicians we interviewed were relatively knowledgeable about the definition and use of antibiotic drugs, there were also noteworthy knowledge gaps about antibiotic resistance and antimicrobial stewardship. However, when provided with a definition of AMS, many of the participants identified steps taken at their current workplaces to implement AMS strategies. Many participants also recognized roles that veterinary technicians could potentially play in AMS efforts. The participants also expressed concern about several barriers, including professional hierarchy and time constraints, that veterinary technicians may face when trying to participate in AMS programs.

The roles and responsibilities of veterinary technicians have expanded in recent years in areas such as dentistry, anesthesia, and exotic animal medicine [17–20]. The results of our study suggest that there is an appetite among veterinary technicians, particularly those in academic or referral practice, to have a greater role in AMS efforts. Many participants reported already playing a key role in infection control, drug administration, and collection of patient medical histories, all of which can impact AMS. Most participants also believed that taking on additional AMS responsibilities could benefit them professionally and empower them within the practice. Studies have suggested that underutilization of training and skills contributes to veterinary technician job dissatisfaction [21, 22] Opportunities to build knowledge and skills, solve complex problems, and have a sense of autonomy have been linked to lower rates of burnout among veterinary technicians [22, 23]. While work hours and salaries are critical drivers of retention, attrition of veterinary technicians is lower among those who feel their work is meaningful and valued [21, 24].

Our study suggests that there are many potential ways that veterinary technicians can further engage with AMS and take on relevant responsibilities. For example, veterinary technicians can play an expanded role in AMS by developing and managing a hospital’s infection control, prevention, and biosecurity program (ICPB). The AVMA considers ICPB a core principle of AMS because these programs may lessen the use of antibiotics by reducing the incidence of disease [2]. The first step of implementing an ICPB program, according to the American Animal Hospital Association (AAHA) guidelines, is to identify a staff member to lead the program [25]. Veterinary technicians are well-suited to this role given their variety of skills, particularly in patient care and record keeping. Veterinary technicians can contribute to ICBP programs in a number of ways, including surveillance for disease, assessment of protocols, and monitoring compliance [25]. Non-ICBP responsibilities related to AMS could also include collection of antibiotic use data, implementation of in-house diagnostic tests, and organization of educational programming or activities.

A majority of participants in this study (25/33) identified education of owners as a way veterinary technicians could play a major role in AMS. Previous studies have demonstrated a need for more owner education in veterinary AMS and a lack of tools for doing so [16, 26–28]. Veterinary technicians are often conduits of information from veterinarians to owners on topics such as nutrition, end-of-life care, and post-surgical home care [29–31]. Examples of AMS topics that could potentially be covered in client-veterinary technician interactions include why antibiotics may not be necessary, adherence to dosing schedule, regimen completion, disposal of drugs, importance of culture and susceptibility testing, and monitoring for adverse reactions. Our study did not specifically assess current veterinary technician knowledge or willingness to engage with clients on these topics and future studies would be needed to determine efficacy of these potential interactions. It is important to note that more than half of the veterinary technicians in this study expressed concerns over clients’ perceptions of them within the hierarchy of the veterinary field (Table 3). Specifically, they expressed concerns that owners may not wish to speak to them about the topic or that they would not take it seriously unless it was communicated by a veterinarian. A study of clients performed in the United Kingdom demonstrated high levels of trust for the information provided by veterinarians but variable levels of confidence in advice provided by veterinary staff and a lack of understanding of their roles and knowledge [32]. In order to engage veterinary technicians in AMS owner education, veterinary hospitals will need to provide training in rapport building and communication skills to their veterinary technicians [32]. Veterinarians could leverage the high degree of trust that clients have in them to promote the expertise and capability of their technical colleagues [33].

Our study demonstrated that most veterinary technicians were able to describe and identify instances of what they believed was inappropriate prescribing of antibiotics (Table 2). Almost all of the participants believed technicians could participate in regular discussion about AMS with veterinarians, but many believed that the hierarchical structures of veterinary practices would be a major barrier. Clearly, therapeutic antimicrobial decisions are the responsibility of the veterinarian, but it is possible that veterinary technicians can provide valuable information to veterinarians when making those decisions, flag instances where therapeutic changes may be needed, or help identify instances of antibiotic misuse as part of AMS programs. Contributions to therapeutic decision-making could be analogous to the role veterinary technicians play in patient pain management [34, 35]. Veterinary technicians are routinely part of monitoring patients for signs of pain or improvement and the effects of pain medications [34]. They use their own clinical knowledge and judgement to make recommendations on approaches to pain management, which are skills that could also be applied to therapy for infection management [35]. It is possible that some veterinarians may not be receptive to feedback from a veterinary technician about antibiotic prescribing, but this may lead to missed opportunities to refine use. Hierarchical structures within the field will need to be addressed to fully engage veterinary technicians in AMS. Structured and open communication between veterinarians and technical staff are hallmarks of effective veterinary teams and could benefit AMS programs [36, 37]. A focus on team effectiveness and empathy have been linked to greater professional quality of life and satisfaction across roles [36, 37].

The other critical barrier identified for veterinary technician AMS engagement was the substantial time constraints of daily practice. Time constraints and staffing shortages are well-documented concerns among veterinary technicians throughout studies of well-being and job satisfaction [21, 22, 24, 38]. Currently, the veterinary profession in the United States is facing a significant shortage in the veterinary technician workforce [39]. As illustrated by a quote in Table 3, participants perceived that the additional time to “stop, slow down and think” about AMS is not likely something that could be changed in practice. Some participants expressed a lack of interest, which when combined with a lack of time would make involvement of at least some veterinary technicians nearly impossible. This potential combination of lack of time and interest means that if additional AMS responsibilities are given to veterinary technicians, they must be afforded ample time to complete them and only given to those who are interested in playing a formal role. This mayrequire a relatively significant reduction in an individual’s role performing traditional clinical duties (e.g. performing anesthesia, collecting diagnostic samples) to make room for AMS specific duties. This, subsequently, would lead to additional strain on other technicians unless that gap is filled by an additional employee or the workload reduced in another manner. Overcoming the barrier of time constraints may be too significant of a burden on a veterinary practice to consider use of veterinary technicians in this role if is simply creates more problems elsewhere.

We found that there is a need to include more education on antimicrobial resistance and stewardship practices in veterinary technician training. Currently, the AVMA Committee on Veterinary Technician Education and Activities (CVTEA) accreditation skills list does not specifically address AMS; however, the list includes many opportunities to highlight AMS throughout veterinary technical progam curricula [40]. The list requires several tasks used in the diagnosis of infectious disease (i.e., collection of samples, performing stains, mastitis testing). The list specifically addresses bacterial culture, speciation, and susceptibility testing as an essential skill. Programs could utilize these opportunities to further engage students on the topic of AMR and provide context of why diagnostics are critical tools in AMS. The skills list also specifically requires that technician students “establish and maintain appropriate sanitation and infection control protocols for a veterinary facility, including patient and laboratory area” which can easily be connected to ICPB and AMS. Finally, specific discussion on the responsible administration, use, disposal, and monitoring of antimicrobial drugs can be integrated into many of the skills listed under Sect. 2 of the “Pharmacology and Pharmacy” list. Veterinary technician programs could also choose to include specific AMS tasks such as “communicate to an owner about the risks of antimicrobial resistance” or “identify an instance of antibiotic misuse.”

Even though this study demonstrated that some veterinary technicians may be willing to engage with AMS responsibilities, there would be many additional challenges to navigate beyond additional basic education. For example, identification of individuals with adequate experience, knowledge and skills to join an AMS team may be challenging particularly without established criteria within the field. Additionally, ability to participate in certain functions (e.g. dispensing pharmaceuticals) may be restricted to only those with certain credentials (e.g. CVT) or could only be performed by veterinarians (e.g. prescribing of drugs, giving advice to owners that would constitute the practice of veterinary medicine) [21–24]. Implementing antimicrobial stewardship programs already requires extreme care and boundaries which may or may not be complicated by adding non-prescriber paraprofessionals to the AMS team. If AMS is to be successful in veterinary medicine, it is important to not hinder efforts to implement these already challenging programs in veterinary hospitals [3, 6, 7].

Some limitations apply to this study. The small number of participants and the overrepresentation of technicians in academic and referral veterinary practices and companion animal medicine limit the generalizability of our results. Specifically, veterinary technicians working within the agriculture sector were not recruited to the study which is appropriate given the distinct regulatory landscape. Most participants were from the northeastern USA which may have some influence on individuals experiences with antimicrobial resistance given variability in resistance patterns. However, what is lost in numbers is gained by the depth of responses of participants and the diversity of perspectives achieved through purposive sampling. Qualitative research, by nature, is meant to be hypothesis-generating, and further research is needed to validate our findings. It is also important to assess the attitudes and opinions of veterinarians about the role of veterinary technicians in AMS. Future directions should include development of specific AMS competencies for veterinary technician students and studies that evaluate the efficacy of training and, ultimately, the impact of the involvement of veterinary technicians in AMS. Finally, throughout our interviews the terms antibiotic and antimicrobial were often used interchangeably by both the interviewer and respondents; therefore, it is possible that some nuance in answers may be lost if participants were considering drugs beyond those with antibacterial activity and this was not explicitly clarified.

The recent but separate efforts to develop veterinary AMS programs and to elevate the professional status of veterinary technicians may be an excellent opportunity for synergy [2, 41]. Our study demonstrates that many opportunities for veterinary technicians exist in AMS and that many technicians would like to contribute. AMS may be an excellent topic to explore as part of veterinary interprofessional education (IPE). The IPE model, which focuses on co-training allied health professionals, has been well-developed in the human health field and has been successfully demonstrated in veterinary medicine [42–44]. Future research should be conducted to understand the opinions of veterinarians on these types of partnerships, and to obtain feedback following their implementation. By working and learning together, both veterinarians and veterinary technicians may be able to have meaningful and lasting impact on veterinary antibiotic prescribing practices and preservation of antimicrobial efficacy.

## Conclusion

Our study demonstrates that many opportunities for veterinary technicians exist within AMS and that many technicians already contribute to AMS. Among some technicians there was an appetite to contribute to AMS more than their traditional roles. If the role of technicians is to grow in AMS, then there is a need to include more education on antimicrobial resistance and stewardship practices in veterinary technician training programs. We also identified several key barriers including time-constraints and professional hierarchy concerns for additional engagement with AMS for technicians. As AMS programs continue to develop in veterinary medicine, it is important to engage the entire clinical team, including veterinary technicians, who may help to ensure AMS program success.

## Methods

We conducted in-depth, semi-structured interviews with a purposive sample of veterinary nurses, technicians, and assistants from different types of hospitals, working with veterinarians from different specialties, and seeing different patient populations. Participants were eligible if they currently worked as a veterinary technician in a clinical setting.

To recruit participants, we worked with ten key contacts at nine veterinary hospitals ranging in size (including two veterinary teaching hospitals) and two contacts at veterinary technician training institutions, including veterinarians, technicians with supervisory roles, faculty at a veterinary technician program or individuals with access to a listserv. These contacts were part of the professional networks of all of the study authors and were selected to participate to represent a wide range of practice types in which veterinary technicians work. Three of the key contacts qualified for the study and were enrolled and the rest shared information with co-workers and other relevant contacts. The study was advertised on the alumni listserv of two local veterinary technician training programs and the author’s institutional veterinary alumni listserv as well as on relevant veterinary social media groups. Additionally, participants were encouraged to share the interview opportunity with other veterinary technician contacts outside of their current place of employment. Veterinary technicians and assistants who were interested in participating underwent screening to ensure eligibility and to maximize the diversity of viewpoints and educational/employment experience among participants.

All interviews were conducted over Zoom which allowed participants to self-identify a location. Interviews were recorded and transcribed. Potential respondents were assured that their specific comments would not be shared with key contacts beyond a report of de-identified aggregated themes. Our protocol was approved by the University of Pennsylvania Institutional Review Board (IRB Protocol # 843,963). Respondents were offered a $25 incentive in the form of a electronic for participation which was deemed not overly influential or likely to result in bias following IRB review.

A semi-structured interview guide (Additional Files) was created based on a review of the literature and the authors’ previous research. Questions were both open- and closed-ended to elicit a variety of both simple and in-depth responses from participants. Key thematic domains in the interview guide included respondent’s work experience and interactions with their employer, perceptions of antimicrobial resistance and overuse in veterinary medicine, the application of principles of AMS within their place of employment, opinions on potential opportunities for veterinary technicians to contribute to AMS and concomitant potential barriers to these opportunities.

Transcripts were reviewed by two authors (LR and KR) and analyzed using conceptual content analysis and a systematic classification process [45, 46] Briefly, all transcripts were initially read individually by both authors, and each author independently identified broad themes. The authors then convened to compare their themes, refined the list to a set of common agreed-upon themes, and organized themes into a hierarchical coding framework. A subset of transcripts was then coded jointly by the authors to ensure consistency and agreement in how codes were applied. The remaining transcripts were coded separately. The presence and frequency of codes was compared across different categories of respondents [47]. QSR NVivo software was used for data coding and categorization.

### Electronic supplementary material

Below is the link to the electronic supplementary material.

Additional File 1: Interview guide

## Data Availability

The datasets used and/or analysed during the current study are available from the corresponding author on reasonable request.

## Abbreviations

AVMA: American Veterinary Medical Association
AMS: antimicrobial stewardship
CVT: certified veterinary technician
CVTEA: AVMA Committee on Veterinary Technician Education and Activities, ICPB:Infection control, prevention, and biosecurity
IPE: Interprofessional education
