# Supplementary material for: The potential role of veterinary technicians in promoting antimicrobial stewardship
Source: BMC Vet Res. 2023 Sep 2;19:142. doi: 10.1186/s12917-023-03637-w (PMC10474764; doi:10.1186/s12917-023-03637-w)
Supplement: Supplementary file 1 — Additional File 1: Interview guide [file 12917_2023_3637_MOESM1_ESM.docx]

Interview Guide

1. **Demographics**

Position title__________________

Age_________

Gender Identity _________

Highest Degree held_____________________

Veterinary Technology Degree Y____ N____

Years as a Vet Nurse_____________

Practice Type _____General ______ Referral ______Shelter

Primary species treated ______________________________________

How many veterinarians do you work directly with? On a given day, and in general________

What kind of interactions do you have with veterinarians in your place of work?

*[Prompt: how often do you interact, what do you talk about, is the veterinarian involved in teaching/mentoring you]*

[If in an academic setting]

What role do you have interacting with veterinary students?

1. **Roles played in Practice**

What are the most common tasks you perform in your role?

Describe the roll you play in and how frequently you:

- Collect patient histories
- Report on patient progress to the veterinarian
  - Specifically about therapeutics
- Calculate the dose of IV drugs and administer IV drugs
- Discuss therapeutic plans with owners
- Participate in infection control measures
- Teach other veterinary nurses?

How often do you interact with pet owners and what kind of interactions do you have with them?

1. **Antibiotic Stewardship**

What, in your own words, is an antimicrobial agent or antibiotic?

[*If participant not clear, provide definition – drug used to treat or prevent bacterial infections]*

How often do you think antimicrobials or antibiotics are prescribed in your practice? For example, on average, out of every 10 patients, how many would receive an antimicrobial or antibiotic? What are the most commonly prescribed antimicrobials or antibiotics in your practice?

What are the most common indications for antibiotics among the patients you see?

What do you think about how antimicrobials are used in veterinary medicine?

*[Prompt: Used appropriately, inappropriately, excessively, not too much, for too long or too short durations]*

In your opinion, what constitutes the inappropriate use of an antibiotic?

Can you think of any examples of inappropriate use of antimicrobials that you’ve seen in veterinary medicine?

What, in your own words, is antibiotic resistance?

Have you ever heard of the term antibiotic stewardship? If so what is it? Where did you first learn about antibiotic stewardship?

*AVMA Definition Antimicrobial stewardship refers to the actions veterinarians take individually and as a profession to preserve the effectiveness and availability of antimicrobial drugs through conscientious oversight and responsible medical decision-making while safeguarding animal, public, and environmental health.In a nutshell, it’s veterinarians and veterinary institutions making conscious efforts to prescribe antimicrobials appropriately to limit antimicrobial resistance.*

With this definition in mind, do you think AM stewardship is happening in your practice? If so, can you provide any examples?

What roles do you believe a veterinary nurse can play in Antimicrobial Stewardship? What would be easy about that? What would be hard?

Have you ever had discussion with your co-workers (other nurses, veterinarians) about antimicrobials and judicious use of antimicrobials?

Would you feel comfortable teaching other vet nurses [and/or students] about antimicrobial stewardship? What messages would you think are important to convey?

Do you think there is an appetite among veterinary nurses to learn about and practice antimicrobial stewardship in veterinary medicine?
